# Opening of the Schools

**Published:** 1873-10-15

**Authors:** 


Opening of the Schools.—The three regular Medical Colleges in this city opened their annual courses of instruction during the first week of the present month. The general introductory lecture in the Rush Medical College was given on the evening of the 1st of October; that in the Chicago Medical College, by Prof. Davis, on the evening of the 6th; and in the Woman's Hospital Medical College, by Prof. A. Fisher, on the evening of the 7th instant. We have not learned the number in attendance, in any, except the Chicago Medical College, which is the Medical Department of the North-Western University. In this, as in all the other departments of the University, the number of students in attendance, at the commencement of this term, is very much larger than in any previous year. The schools, hospitals and dispensaries of Chicago, at the present time, afford as complete and abundant facilities for education, in every department of medicine, as those of any city on this continent.

